# Prognostic Biomarkers and EBV Infection Research in Diffuse Large B-Cell Lymphoma of the Palatine Tonsils

**DOI:** 10.5402/2012/652682

**Published:** 2012-02-16

**Authors:** Marinho Marques, Estela Luz, Michael Hummel, Maria das Graças Vieira, Regina Célia Bahia, Maria Cristina Oliveira, Eduardo Martins Netto, Ivana Luz, Iguaracyra Araújo

**Affiliations:** ^1^Serviço de Hematologia, Núcleo de Oncologia da Bahia, Avenida Adhemar de Barros 123, Ondina, 40170-110 Salvador, BA, Brazil; ^2^Hospital Universitário Professor Edgard Santos, Universidade Federal da Bahia, Rua Augusto Viana s/n, 40110-060 Salvador, BA, Brazil; ^3^Institute of Pathology, Charité Universitätsmedizin Berlin, Campus Benjamin Franklin, Hindenburgdamm 30, 12200 Berlin, Germany; ^4^Serviço de Hematologia, Hospital Aristides Maltez, Avenida D. João VI 332, Brotas, 40285-001 Salvador, BA, Brazil

## Abstract

Diffuse large B-cell lymphoma represents approximately 30%–40% of all diagnoses of non-Hodgkin's Lymphoma and may represent up to 80% of all lymphomas that arise in the palatine tonsils. Several studies have attempted to correlate clinical, laboratorial, and tissue factors with the prognosis of the lymphomas, such as the International Prognostic Index, the tissue expression of some proteins, and the lymphocyte count at the time of diagnosis, as well as to correlate Epstein-Barr virus (EBV) infection with worse prognoses. Patients with palatine tonsil DLBCL, from Salvador, Bahia, Brazil, were studied in order to identify prognostic factors. Twenty-four patients with DLBCL were studied. The factors that negatively influenced the patients' survival rates were the lymphocyte count at the time of diagnosis <1.000/mm^3^ and the Bcl-2 protein expression. There was no CD5 expression in these lymphomas, and neither was there an association with EBV infection.

## 1. Introduction

The palatine tonsils, along with the nasopharyngeal lymphoid tissue, the base of the tongue, and the oropharyngeal wall make up Waldeyer's ring. This ring is located at the entrance of the respiratory and digestive tract, being the second most common site of extranodal lymphomas, after the gastrointestinal tract [1, 2]. These tumors represent 15 to 20% of all lymphomas and half of the head and neck lymphomas. Approximately 50% of Waldeyer's ring lymphomas arise in the palatine tonsils in presentation, and in approximately 20% of the cases they are bilateral [3].

Most lymphomas found in the palatine tonsils are the B-cell type, and, of these, diffuse large B-cell lymphoma (DLBCL) represents most of the cases, reaching as much as 80% in some of the groups studied [3, 4].

Although morphologically indistinct, some molecular studies support the hypothesis that DLBCL makes up a heterogeneous group of lymphomas that has different prognostic implications [5]. Classically, the International Prognostic Index (IPI) has been used to predict the survival of patients with DLBCL [6]; however, it is not useful in all cases. Studies using DNA microarray analysis show that the DLBCL gene expression profile similar to B cells germinal center would have a better clinical prognosis than the profile similar to activated B cells [5, 7]. Transposing this gene profile to a protein expression, Hans et al. [8] proposed an algorithm to classify DLBCL patients, using three immunohistochemical markers (CD10, Bcl-6, and MUM1). The profile that was similar to the germinal center (GC) presented a better survival rate than the profile of the nongerminal center (non-GC). Other tissue markers, such as Bcl-2, CD5, and p53 protein have been referred to as prognostic predictors in DLBCL [9–11]. A lymphocyte count <1.000 cells/mm³ at the time of diagnosis has recently been described as associated with a worse prognosis in DLBCL [12, 13].

Another controversial prognostic tissue marker has been the detection of Epstein-Barr virus (EBV) infection in neoplastic cells. While in pediatric Hodgkin's Lymphoma this infection is associated with a better prognosis [14], in adults the infection in DLBCL seems to be associated with a worse prognosis [15]. EBV belongs to the herpes virus family, is transmitted through contact with saliva, and infects mostly B lymphocytes and occasionally oropharyngeal epithelial cells. Serum-epidemiological studies show that more than 90% of the patients with DLBCL, worldwide, are infected with EBV [16]. This infection has been detected in approximately 9% of patients with DLBCL [15]. However, in head and neck lymphomas, this index seems to be higher, reaching 90% in Egypt [17]. In Brazil, this infection has been associated with pediatric lymphomas originating in germinal center cells [18], although the index of this infection in adults with DLBCL has not yet been studied.

Considering that the use of IPI has been shown to be insufficient as the only prognostic marker in DLBCL, we evaluated other laboratory and tissue markers in patients with DLBCL of palatine tonsils, who came from a reference institution for the diagnosis and treatment of patients with cancer in Northeastern Brazil.

## 2. Patients and Methods

### 2.1. Patient Selection

The patients were selected at the Pathological Anatomy Service of the Aristides Maltez Hospital in Salvador, Bahia, Brazil. All patients with a diagnosis of DLBCL of the palatine tonsils were included. The diagnoses were carried out between January of 1999 and December of 2006, on patients with illness primary site in the tonsils, or on those who presented the main tumor mass in the same area.

The diagnoses were reviewed by a hematopathologist (I.A.) according to the criteria established by the lymphoid neoplasia classification of the World Health Organization 2008 [19]. The clinical data were gathered from medical chart records, and patients with incomplete medical charts were excluded. The staging was obtained using the Ann Arbor criteria [20], and the IPI was obtained according to previously established parameters [6]. This current work was approved by the Ethics Committee for Human Subjects Research and complies with the principles of the Helsinki Declaration.

### 2.2. Immunohistochemical (IHC) Study

The tissue used in the study had been set in formalin. Serial 4 *μ*m sections of tissue blocks were mounted on silanized slides, deparaphined with xylene, and cleansed in alcohol. The streptavidin-biotin-peroxidase technique was used, with previous, humid heat antigen recovery. A commercially available panel of antibodies was used (Table 1).

For the antibodies CD3, CD5, CD10, CD20, Bcl-2, Bcl-6, and MUM1, the cases with more than 10% of the tumor cells marked were considered positive. For the Ki-67 analysis, the cases were semiquantified on a positivity scale of 0 to 100%, according to the quantity of marked tumor cells per field, in an increase of 400x. The p53 protein analysis was carried out using the Sannino and Shousha score [21].

The IHC classification according to the Hans et al. algorithm [8] in GC and non-GC profile was carried out using the C10, Bcl-6, and MUM1 markers. Patients who were positive for CD10 alone, or positive for CD10 and Bcl-6, were considered as GC profiles. Patients who were negative for CD10 and Bcl-6 were considered as non-GC profiles. Patients who were negative for CD10 and positive for Bcl-6 were classified after the MUM1 analysis. The cases that were negative for MUM1 were classified as GC profiles, and the positive cases were classified as non-GC profiles.

### 2.3. In Situ Hybridization

In situ hybridization for the detection of EBER 1 and 2 transcription of EBV was carried out in RNA-free conditions, using specific probes marked with digoxigenin, as previously described [18]. For positive controls, tissue from patients with Burkitt lymphoma and infectious mononucleosis was used, previously identified as positive for EBV. The sign considered positive was located in the nucleus.

### 2.4. Statistical Analyses

To compare the difference between the two proportions, the chi-square test and Fisher exact test were used. The differences between two means were analyzed by the Mann-Whitney test. The Kaplan-Meier survival analysis and the log-rank test were used to study the prognostic significance of the utilized biomarkers. The overall survival (OS) was calculated from the date of diagnosis to the last evaluation, or date of death. Event-free survival (EFS) was calculated from the date of diagnosis until date of death, disease progression, or end of clinical followup. The *P* value was considered significant when <0.05. The SPSS software version 16.0 was used to carry out all the calculations.

## 3. Results

### 3.1. General Characteristics of the Patients

During the time of the study 567 diagnoses of non-Hodgkin's Lymphoma were made in the study institution. Of these, 253 were classified as DLBCL (44.6%). Twenty-six diagnoses of DLBCL of the palatine tonsils were made (4.6% of the total amount of DLBCL), and for this present work 24 cases of DLBCL of the palatine tonsils were studied, due to the exclusion criteria.  Table 2 shows the characteristics of the study patients.

Most of the patients were treated with a schema based on anthracycline (CHOP or similar ones). The average follow-up time of the patients was 43 months (range 1 to 104 months). The overall and event-free survival rates were on average of 43.5 months to 39.5 months, respectively. Most of the patients presented a low-risk IPI (Table 2), and, if compared to patients with none or 1 factor versus >1 factor, this index did not significantly influence the OS (71.6 months versus 31 months, *P* = 0.30, resp.) and the EFS (71.2 months versus 30 months, *P* = 0.26, resp.) of the patients.

### 3.2. Lymphocyte Count at the Time of Diagnosis

**Figure 1:**
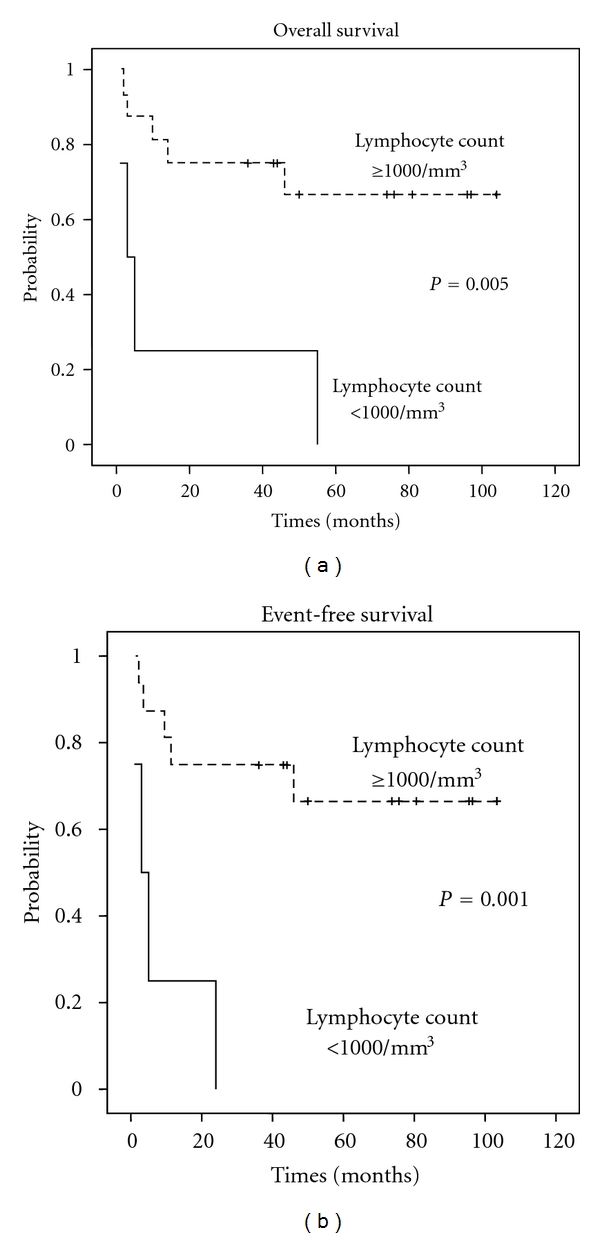
Overall survival and event-free survival considering lymphocyte count at the time of diagnosis.

The mean lymphocyte count at the time of diagnosis was 1.980 cells/mm³, varying between 354 and 3.922 cells/mm³, and 18.2% of the patients presented a lymphocyte count <1.000 cells/mm³. Patients with a lymphocyte count at the time of diagnosis ≥1.000 cells/mm³ presented OS (74.9 months versus 16 months) and EFS (74.7 months versus 8.2 months) significantly greater than patients with a count lower than this value (*P* = 0.005 and *P* = 0.001, resp.) (Figure 1).

### 3.3. Immunohistochemistry

**Figure 2:**
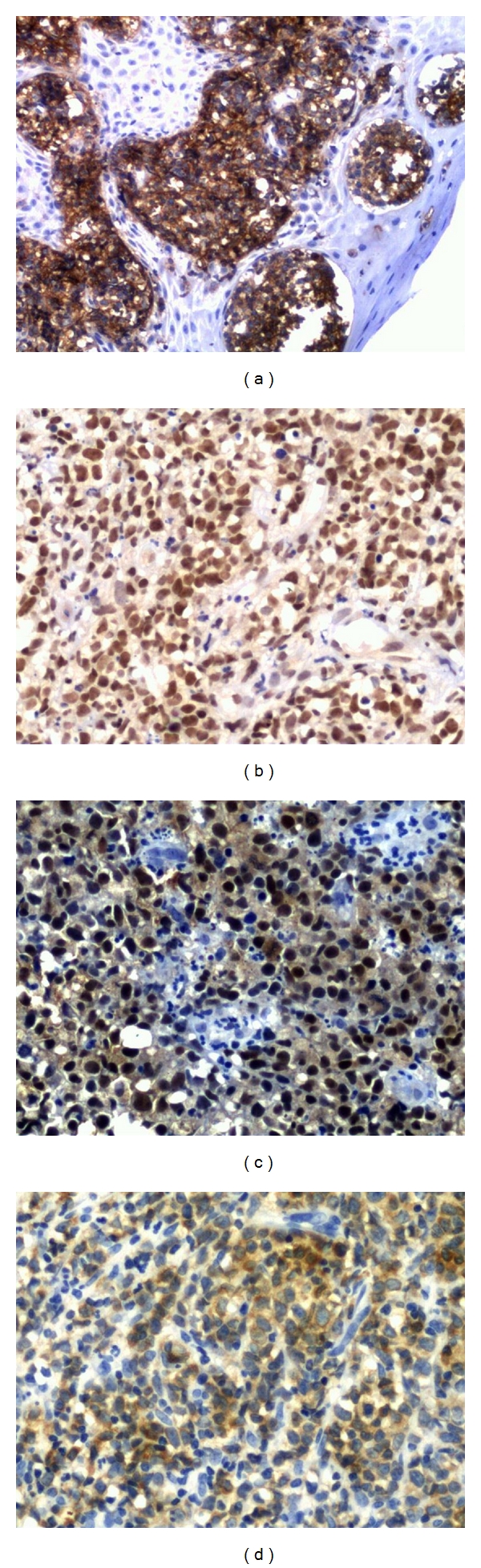
Immunohistochemical aspects of the palatine tonsils DLBCL: positivity for CD10 (a), Bcl-6 (b), MUM-1 (c), and Bcl-2 (d) antibodies.

According to the algorithm of Hans et al. [8], 10 patients were classified with GC profile (41.6%), and 14 were classified with non-GC profile (58.4%). Patients with GC profile presented better OS (64.2 versus 46.5 months) and EFS (63.5 months versus 46.1 months) than patients with non-GC profile, although with no statistical significance (*P* = 0.36 and *P* = 0.33, resp.). The isolated positivity for the markers used in the Hans algorithm (CD10, Bcl-6 and MUM-1, Figures 2(a), 2(b) and 2(c), resp.) also did not have significant influence on patients' survival rates.

**Figure 3:**
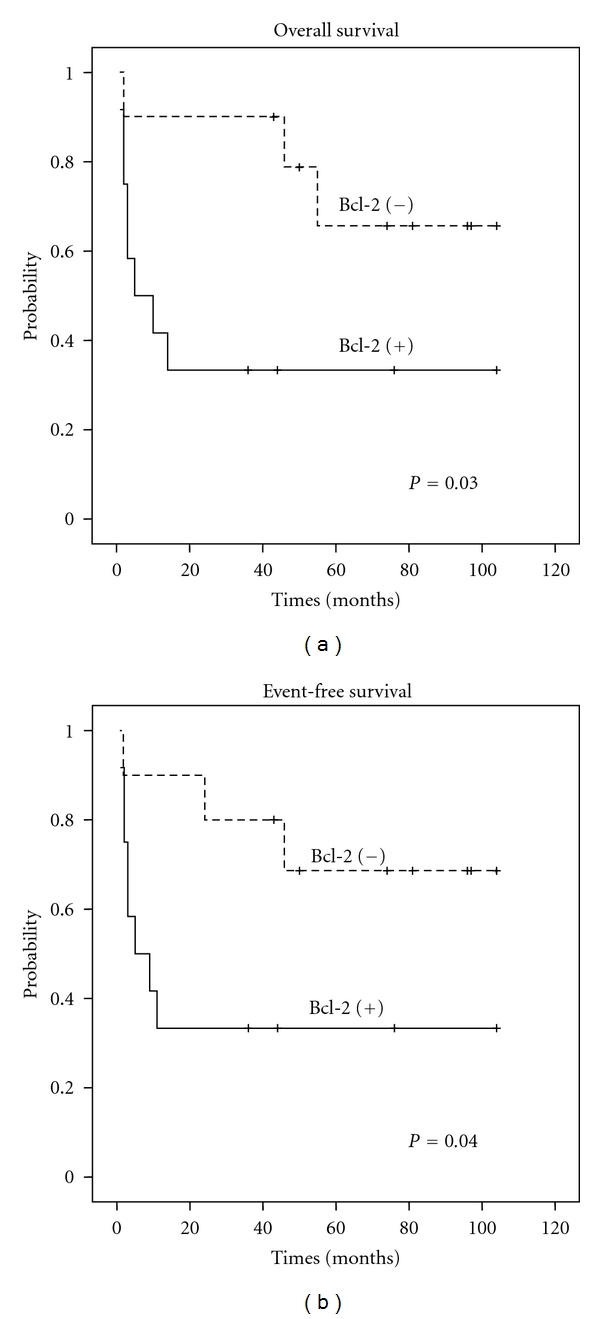
Overall survival and event-free survival considering Bcl-2 protein status at the time of diagnosis.

The Bcl-2 protein expression (Figure 2(d)) was found in 54.2% of the patients, and these were significantly older than the patients who were negative (65 versus 44 years old, resp., *P* = 0.02). Patients who were positive for Bcl-2 presented significant worse OS (38 months versus 80.8 months, resp.) and EFS (37.6 months versus 79.1 months, resp.) rates than negative patients (*P* = 0.03 and *P* = 0.04, resp. (Figure 3)).

The p53 protein expression was found in 45.8% of the patients with predominance of the male gender (*P* = 0.03) and young patients (mean age of 49 years in positive patients versus 63 years in negative patients, *P* = 0.08). Patients with p53 protein expression presented greater OS and EFS rates than negative patients (68.6 months versus 43.3 months and 67.1 months versus 43.3 months, resp.), but there was no statistical significance (*P* = 0.22 and *P* = 0.26, resp.). The mean Ki67 was 60%, varying from 30 to 100%. There was no CD5 expression in any of the patients studied.

### 3.4. In Situ Hybridization

All the patients presented negative in situ hybridization for EBER-1 and 2 transcription of EBV.

## 4. Discussion

Among the non-Hodgkin's lymphomas, we observed a DLBCL frequency similar to other studied series; however, the palatine tonsils were mostly attacked at a slightly greater frequency than that referred to in the literature [1, 2]. Similar to other studies, we observed a predominance of B lymphomas (mainly DLBCL) in this site, as well as more advanced age at the time of diagnosis, around 60 years [22].

We observed a predominance of tonsil lymphoma patients and low-risk IPI. When observed alone, the IPI did not show any significance to predict survival rates, and this index might not be the most adequate prognostic factor for patients with extranodal lymphoma. However, the reduced number of patients in this study may have had an influence on this analysis.

Recent studies have shown the lymphocyte count at the time of diagnosis with a prognostic factor in DLBCL [12, 13], as already shown in other hematological neoplasias such as Hodgkin's lymphoma [23], follicular lymphoma [24], and acute myeloid leukemia [25]. In the current study, this correlation was also observed, for patients with a lymphocyte count ≥1.000 cells/mm³ at the time of diagnosis presented significantly greater OS and EFS rates (*P* = 0.005 and *P* = 0.001, resp.).

DLBCL has several forms of presentation and is a heterogeneous entity seen as certain IHC marker expressions, which could give it a better or worse prognosis. Hans et al. [8] observed that patients classified as GC profile (using an algorithm that regards the expression of C10, Bcl-6, and MUM1 for classification) presented better survival rates than patients with non-GC profile. Patients, in this study, who had tonsil lymphoma and GC profile, were observed as having better survival rates; however, a statistical significance was not reached, possibly due to the small number of patients. The isolated expression of algorithmic immunohistochemical markers did not show a correlation with survival rates.

In a more consistent manner, the Bcl-2 expression in the pre-Rituximab era has proven to be an unfavorable prognostic factor [9]. In the present study this finding was confirmed, for this protein expression was linked to worse GS and EFS rates. In these patients, the Bcl-2 expression was present in a significant way in older patients (*P* = 0.02), which might have influenced OS and EFS rates negatively due to the combination of both factors.

Still quite a controversial issue is the prognostic value of the p53 protein expression in patients with DLBCL. In two other Brazilian studies that researched this correlation [26, 27], only one was able to show a significant difference in OS [26]. In this current study, positivity was associated with better OS (with no statistical significance), and the patients who were positive for p53 tended to be younger, which might have influenced this observation.

In the present study all the patients were negative for the CD5 marker. The expression of this marker has been reported as between 5%–10% of the patients who have *de novo* DLBCL, granting a worse prognosis [10]. Due to the negativity we were unable to evaluate this data. However, in another study involving patients with DLBCL of the respiratory-digestive tract, the 17 patients with palatine tonsil lymphoma were also negative for CD5 [28], similar to the findings of this study.

Several studies in the literature have demonstrated the association between EBV infection and some subtypes of non-Hodgkin's lymphoma [15, 17]. In the patients with diffuse large B-cell lymphoma, this association is undergoing confirmation, with the appearance of many studies seeking to reinforce the association with EBV. There is even a correlation with the prognosis of these lymphomas, which is an unfavorable prognostic factor in EBV-positive patients, presenting worse treatment response, as well as worse overall survival, and event-free survival rates [15].

No association was found between EBV infection and DLBCL in the patients of this study. This data is different from what was found by Park et al., who studied 380 patients with DLBCL, where 9% presented EBV infection [15]. Also, Bahnassy et al. showed EBV infection in 90% of the fifty cases of head and neck NHL studied in Egypt [17]. Although it was shown previously that the tonsils of Brazilian children had significantly more EBV-infected cells than the tonsils of German children [18], the negativity for EBV infection was found by Wong et al. when they studied 17 patients with palatine tonsil DLBCL in Malaysia [28].

We conclude that, in our midst, the palatine tonsil diffuse large B-cell lymphomas predominantly presented a non-GC profile and were not associated with EBV infection. The factors that negatively influenced the OS and EFS rates of these patients were the lymphocyte count at the time of diagnosis <1.000/mm³ and the Bcl-2 protein expression. New studies are necessary to attempt to explain the etiology of these lymphomas and to identify other prognostic factors.

## Figures and Tables

**Table 1:** Antibodies used for immunohistochemical study.

Antibody	Clone	Dilution	Source
CD3	F7.2.38	1 : 100	DakoCytomation
CD5	4C7	1 : 50	Novocastra
CD10	56C6	1 : 50	Novocastra
CD20	L26	1 : 100	DakoCytomation
Bcl-2	124	1 : 50	DakoCytomation
Bcl-6	P1F6	1 : 20	Novocastra
MUM1	MUM1p	1 : 50	DakoCytomation
p53	PAb1801	1 : 100	Novocastra
Ki67	MIB-1	1 : 50	DakoCytomation

**Table 2:** Clinical features of the patients.

Parameters	Frequency (%)
Gender	
Male	11 (45.8)
Female	13 (54.2)
Age (mean)	60 (range 15–86)
Stage	
I/II	13 (45.8)
III/IV	6 (25)
B symptoms	12 (50)
Performance status	
<2	23 (95.8)
≥2	1 (4.2)
Serum LDH	
Normal	16 (66.7)
Elevated	2 (8.3)
IPI	
0 and 1	15 (62.5)
2	2 (8.3)
3	1 (4.2)
4 and 5	0

LDH: lactate dehydrogenase; IPI: International Prognostic Index.
